# A comprehensive systematic review of stakeholder attitudes to alternatives to prospective informed consent in paediatric acute care research

**DOI:** 10.1186/s12910-018-0327-9

**Published:** 2018-11-20

**Authors:** Jeremy Furyk, Kris McBain-Rigg, Bronia Renison, Kerrianne Watt, Richard Franklin, Theophilus I. Emeto, Robin A. Ray, Franz E. Babl, Stuart Dalziel

**Affiliations:** 1 College of Public Health, Medical and Veterinary Sciences, James Cook University, Townsville, QLD 4814 Australia; 2 University Hospital Geelong, Geelong, Victoria Australia; 3 Townsville Health Library, Townsville Hospital and Health Service, James Cook University, Townsville, QLD Australia; 4 Research Methods & Injury Epidemiology, College of Public Health, Medical and Veterinary Science, James Cook University, QLD, Townsville, 4811 Australia; 5 College Medicine and Dentistry, James Cook University, Townsville, QLD Australia; 6 Emergency Department, Royal Children’s Hospital, Melbourne, 50 Flemington Rd, Parkville, VIC 3052 Australia; 7 Murdoch Children’s Research Institute, Melbourne, 50 Flemington Rd, Parkville, VIC 3052 Australia; 8 Department of Paediatrics, Faculty of Medicine, Dentistry and Health Sciences, University of Melbourne, Melbourne, Grattan St, Parkville, VIC 3010 Australia; 9 Emergency Medicine Research, Children’s Emergency Department, Starship Children’s Hospital, Auckland District Health Board, Private Bag 92024, Auckland, 1142 New Zealand

**Keywords:** Consent, Paediatrics, Emergency care

## Abstract

**Background:**

A challenge of performing research in the paediatric emergency and acute care setting is obtaining valid prospective informed consent from parents. The ethical issues are complex, and it is important to consider the perspective of participants, health care workers and researchers on research without prospective informed consent while planning this type of research.

**Methods:**

We performed a systematic review according to PRISMA guidelines, of empirical evidence relating to the process, experiences and acceptability of alternatives to prospective informed consent, in the paediatric emergency or acute care setting. Major medical databases and grey sources were searched and results were screened and assessed against eligibility criteria by 2 authors, and full text articles of relevant studies obtained. Data were extracted onto data collection forms and imported into data management software for analysis.

**Results:**

Thirteen studies were included in the review consisting of nine full text articles and four abstracts. Given the heterogeneity of the methods, results could not be quantitatively combined for meta-analysis, and qualitative results are presented in narrative form, according to themes identified from the data. Major themes include capacity of parents to provide informed consent, feasibility of informed consent, support for alternatives to informed consent, process issues, modified consent process, child death, and community consultation.

**Conclusion:**

Our review demonstrated that children, their families, and health care staff recognise the requirement for research without prior consent, and are generally supportive of enrolling children in such research with the provisions of limiting risk, and informing parents as soon as possible. Australian data and perspectives of children are lacking and represent important knowledge gaps.

**Electronic supplementary material:**

The online version of this article (10.1186/s12910-018-0327-9) contains supplementary material, which is available to authorized users.

## Background

There is a community expectation that children presenting to emergency departments (ED) and acute care settings receive the best possible care based on high-level evidence. The reality though is many treatment decisions are not evidence based, but rather based on theoretical considerations, simply reflecting “what we have always done” or extrapolated from adult data [1, 2]. This is inappropriate as children differ from adults both anatomically and physiologically and health conditions may be entirely unique to the paediatric population [3]. Clinical research in children is necessary for paediatric emergency medicine to advance.

The ethical issues involved in the conduct of paediatric clinical research are complex and are compounded in time critical and life threatening situations in emergency care. The guiding principles of conducting ethical research are: respect for autonomy, beneficence and justice [4]. Respect for autonomy is usually reflected in obtaining informed consent from participants, which remains a fundamental principle in the protection of human participants in medical research. When the participant is a child, consent must usually be obtained from a parent or proxy. While proposing to conduct research without informed consent may seem to contravene the ethical principle of respect for autonomy, denying participation in research to those unable to consent contravenes the ethical principle of justice, meaning fair distribution to the benefits of research participation and fair access to the benefits of research [4, 5].

Children are usually considered a “vulnerable” group in terms of participation in research due to their inability to consent and potential for exploitation [4]. While not without controversy, emergency research without consent has been performed in adults for some time; it is relatively less established in paediatric emergency and critical care. Emergency patients themselves are often considered a vulnerable group, given their reliance on the care being offered [6]. Thus research conducted on children in the emergency setting leaves participants vulnerable on multiple counts.

Performing clinical research in emergency settings is difficult. The environment is often chaotic and unpredictable, presentations of interest may be rare in individual institutions, staff are often stretched with clinical responsibilities, and interventions may have a narrow therapeutic window. One of the many challenges researchers face in conducting research in the ED and other acute care settings is the difficulty of obtaining prospective informed consent [7–9]. Valid prospective informed consent requires provision and comprehension of information about the purpose, methods, demands, risks, inconveniences, discomforts and possible outcomes of the research [4]. In Australia this assumes the capacity for decision-making, a free and voluntary process including adequate disclosure regarding the act performed. Several of these components may not be possible in time critical situations in the acute care setting and there may be an argument for a waiver of informed consent, retrospective or deferred consent. A waiver of informed consent refers to research that has ethical approval to proceed without the requirement for participant or proxy informed consent. Deferred or retrospective consent describes a process where participants are enrolled without informed consent, followed by requesting permission to continue in the study, or if the study intervention has ended, permission to use the data [1].

Guiding principles for use of alternatives to prospective informed consent in emergency research are outlined in the Declaration of Helsinki; “if the research cannot be delayed, the study may proceed without informed consent provided that the specific reasons for involving subjects with a condition that renders them unable to give informed consent have been stated in the research protocol and the study has been approved by a research ethics committee. Consent to remain in the research should be obtained as soon as possible from the subject or a legally authorized representative” [10]. These principles are further reiterated in local documents such as the National Health and Medical Research Council (NHMRC) National Statement on ethical conduct in human research, which allows consent to occur after an intervention if consent is not practicable, there is potential benefit to the child, risk is low, the research has merit and there is no reason to suspect the parents would not give consent. Similar requirements exist in New Zealand [6], the United Kingdom (UK) [11], and the United States of America (USA) [12]. Although implementation is variable, and specific requirements differ internationally, most require the research to be “therapeutic” rather than “non-therapeutic”, offering potential benefit to the participant and pose no more than “minimal risk” [7, 13].

The ethical issues of paediatric acute care research are complex. Even if the therapeutic window of the intervention allows an informed consent discussion and a proxy is immediately available, parents may not have capacity to undertake such decisions. There may be the perception of coercion to participate in research by parents who are dependent on receiving emergency care for their children. Locally, ethics guidance documents such as the NHMRC national statement lack clarity regarding specific requirements for research in these circumstances, and are variably interpreted by ethics committees. There is a paucity of evidence of the acceptability of research without prospective informed consent in paediatric acute care. It is important to explore and understand the perceptions and experiences of parents, health care workers and researchers to alternatives to prospective informed consent in paediatric acute care and emergency research to inform the design of future research and guidance documents.

### Aim/objective

This paper aims to review and synthesize the available empiric evidence with regard to alternatives to prospective informed consent in the context of paediatric acute care research from the perspective of the children, their families, health care staff, institutions, and the community.

## Methods

We performed a comprehensive systematic review according to the Preferred Reporting Items for Systematic Reviews and Meta-Analysis (PRISMA) guideline [14].

### Search strategy

The literature search was designed in conjunction with a medical librarian (BR) and included major databases: Medline (Ovid), Embase (Ovid), Web of Science, CINAHL, and PsycINFO. No limits were set with regard to language or date restriction. See Additional file 1 for Medline (Ovid) search strategy. The electronic database search was run in April 2017 and updated in Jan 2018.

The database search was supplemented by a Google Scholar search using the “cited by” feature, and a grey literature search including conference proceedings, government reports, raw data, theses and dissertations using the key words identified for searching medical databases. Conference abstracts of key recent emergency medicine meetings were hand searched for additional studies. A manual search was conducted of reference lists from identified articles.

### Registration

The review was prospectively registered on the PROSPERO registry for systematic reviews. (PROSPERO 2016 CRD42016053963).

### Study selection

Studies identified by the search strategy were exported into an EndNote library and duplicates removed. Title and abstracts were reviewed independently by two authors (JF and KM), and assessed against eligibility criteria. Disputes were resolved with discussion, and adjudication by a third author (RR).

### Inclusion/exclusion criteria

All study types (quantitative, qualitative and mixed methods) reporting original, empirical evidence relating to the process, experiences and acceptability of alternatives to prospective informed consent, in the paediatric, emergency or acute care setting were included. Perspectives of participants, parents or caregivers, clinicians, researchers and other staff were considered relevant. Studies reported in abstract only were considered. Studies conducted in the pre-hospital environment, emergency department and intensive care unit within all cultural and geographical contexts were included.

Studies that did not present original data e.g. reviews, commentaries, editorials, opinion pieces and letters to the editor were excluded. Studies conducted in the Neonatal Intensive Care Unit were excluded, as these units have their own unique clinical and ethical considerations, which were beyond the scope of this review. Studies only reporting adult patient data, or if paediatric subgroups were not reported separately, were excluded. Quality assessment was performed and reported; however study quality was not a selection criterion.

### Data extraction

Data extraction was performed independently by two authors (JF and KM), and consisted of demographic details of the population studied, phenomenon of interest, methods used, main findings, and conclusions of the authors etc. Data extraction was an iterative process, and new emerging themes were crosschecked with primary articles.

### Data analysis and synthesis

Identified full text studies and data extraction forms were imported into NVivo 11 for Mac for analysis (NVivo qualitative data analysis Software, QSR International Pty Ltd., version 11.1: 2016). We used an inclusive approach to data extraction, with all potentially relevant data included in the synthesis. Text from primary articles was coded into themes using the software. Primary themes identified from general background literature and reviews on alternatives to informed consent from adult literature formed the baseline analysis, and new themes iteratively added during analysis. The validity of the data extraction was reviewed by other authors (KW, RR, TIE).

We used thematic synthesis to synthesize results of our review, which involved free coding of textual data from primary studies, organization into descriptive themes, and generation of analytical themes producing a new interpretation. This technique is similar to meta-ethnography and grounded theory and is useful when drawing together common elements in heterogeneous studies [15, 16].

### Critical appraisal of included studies

Quantitative observational studies were assessed using the “Quality Assessment Tool for Observational Cohort and Cross-Sectional Studies” from the National Heart, Lung and Blood Institute [17]. For qualitative studies we used the “Qualitative Assessment and Review Instrument” (QARI) developed by the Joanna Briggs Institute [18]. The assessment was made by two authors independently (JF and KM), by extracting the relevant text from the publication that addressed the quality assessment criteria, and assigning each question yes, no, unclear or not applicable as to whether quality criteria was met. Disagreements were resolved by consensus or by consulting with third author (RR).

Studies were not excluded on the basis of this assessment as there is no empirically tested method of exclusion of such studies on the basis of quality. Sensitivity analysis was performed excluding studies globally assessed as “poor quality” to determine to what extent exclusion of these studies affected the review e.g. if excluding themes generated from the original synthesis affects the “thickness” of detail in the synthesis.

### Rigor

Methodological quality was ensured by a process coding by multiple authors and triangulation with disputes resolved by consensus.

## Results

**Fig. 1:**
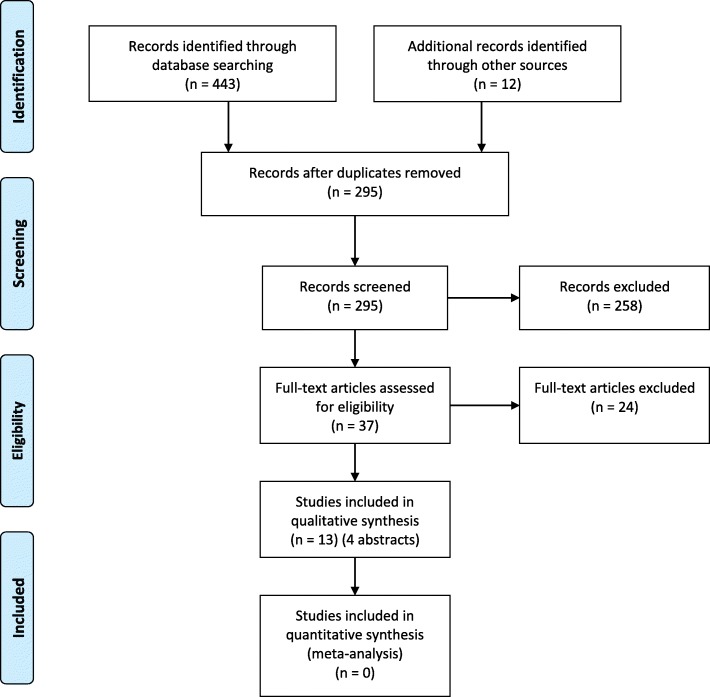
PRISMA flow diagram

The search identified 443 studies (CINAHL 30, Embase (Ovid) 227, Medline (Ovid) 156, PsycINFO (Ovid) 9, Web of Science (21), leaving 295 after removal of duplicates. An additional 12 articles were identified from other sources including reference lists, cite feature and Google scholar. A review of titles and abstracts resulted in 37 articles for full text review. Of these 24 studies were excluded, five studies published as abstracts were duplications of subsequently published full text articles, five abstracts and 14 other studies were excluded as they did not meet inclusion criteria. This is summarised in Fig. 1. Thirteen studies were included in the review consisting of nine full text articles and four abstracts.

Characteristics of included studies are summarised in Tables 1. Critical appraisal of included articles is summarised in Tables 2 and 3. Critical appraisal of the four studies included in abstract form was not possible. Given the heterogeneity of the methods, results could not be quantitatively combined for meta-analysis. Qualitative results are presented in narrative form, according to themes identified from the data.

**Table 1:** Characteristics of included studies

Author/year	Setting	Phenomenon of interest	Study Design	Participants	Key findings	Comments
Bulger EM, 2009[26]	U.S. 5 urban centres (Dallas, Milwaukee, Portland, San Diego, Seattle)	Description of community consultation process for planned RCT	Random dialling survey	500 participants	A majority of subjects indicated a willingness to participate in the study should they become eligible. The average cost of surveys was US$15,000 per site.	Only a small portion data relevant to paeds. Proposed trial was adults, but included questions on children aged 15–17 years
Gamble C, 2012[27]	UK, proposed RCT with deferred consent	Parental perspectives on proposed use of deferred consent in double blind RCT	Postal survey	68 parents and families, 19 (28%) bereaved, members of the Meningitis Research Foundation (MRF).	Deferred consent was generally acceptable. Death of a child posed a uniquely difficult situation. Communication should be flexible and responsive to needs of parents	
Harron K, 2015[20]	U.K. 12 NHS trusts, RCT (CATCH trial)	Consent rates as a percentage of those approached, and those for whom consent was obtained.	Descriptive study of consent rates in RCT utilising varying methods of consent	1859 children included in CATCH trial	1859 children included in CATCH, 1358 (73%) admitted on an emergency basis. Families approached for DC 1178/1358 (87%) of emergency admissions (remaining 180 (13%) not included analysis). Inclusion rates differed according to whether the child died or survived	
Holsti, 2015[32]	15 sites in U.S and Canada. Academic paediatric emergency departments	Effectiveness of community consultation and public disclosure activities	Mixed methods. Survey of participants.	297 enrolled participants completed de-briefing form.	Activities varied widely among sites. Median time from protocol release to final IRB approval was 10 months. Focus groups not associated with another meeting were not well attended. Median cost of $6989.	
Molyneux 2013[21]	Kenya and Uganda, RCT	Staff and parental perceptions of the consent process	Qualitative study of parents and staff	34 interviews with parents of participants, 12 interviews with parents of admitted non-participants, 30 staff interviews. Two focus groups of health workers, 6 interviews with hospital managers.	The DC process with prior assent was supported. Prior assent was seen as protecting the interests of both patients and researchers, including through minimising delays in starting treatment.	
Morris, 2004[19]	U.S. PICU setting	Attitudes to exception to informed consent in a clinical trial	Qualitative study	Focus groups. Parents of children resuscitated from cardiac arrest *n* = 12, parents of children in PICU *n* = 11, PICU nurses *n* = 13, physicians n = 10, administration *n* = 10	Concluded prospective informed consent was not feasible and endorsed exception of IC if have an explicit opportunity to decline participation	Proposed intervention was to be instituted within 30 min, therefore had more time that other scenarios
Scholefield, 2013[31]	28 UK EDs	“appropriateness” of DC	Web-based survey	77 Emergency Medicine consultants	74% approved the use of deferred consent in such a trial.	Limited data relevant to review question (single question)
Stanley, 2017[24]	16 level I paediatric trauma centre EDs in USA	Describe the clinical characteristics, and timing of parent guardian arrival.	Prospective, observational study	295 children with blunt head trauma with Glasgow Coma Scale (GCS) scores of 3–12 (i.e., moderate-to-severe TBI).	The timing of patient and guardian arrival posed a challenge for timely enrolment. The Federal Exception from Informed Consent for Emergency Research is an important consideration for planning such research.	Limited data relevant to review question
Woolfall, 2013[22]	UK (clinical trial units)	experiences and attitudes of practitioners (doctors and nurses) involved in recruiting to clinical trials in the EC setting.	Semi-structured questionnaire	16 consultant grade doctors, 29 research nurses (purposeful sampling)	Views on DC differed with experience with the consent method. Practitioners with no experience reported negative perceptions, with concerns about the impact on the parent-practitioner relationship. Practitioners experienced in DC described how families were receptive to the consent method, if conducted sensitively at an appropriate time.	
Woolfall, 2014[28]	UK (in setting of planned RCT)	To explore the views of parents on proposed RCT i.e. approach to seeking DC and content of PIS.	Qualitative study	17 parents (11 telephone interviews) 6 in focus groups. Purposefully sampled from support groups with acute and chronic conditions.	Most supported DC to enable progress of emergency care research. The child’s safety was a priority and parents were reassured interventions under investigation are both used in routine clinical practice. Parents made recommendations on the need to individualise approaches bereaved parents.	Limitation – low participation rate.
Woolfall, 2015[23]	UK multicentre, (setting of RCT CATCH)	Parents’ views and experiences of the CATCH trial recruitment, the consent seeking procedures and decision-making	Mixed method study (survey, interview and focus groups)	275 parents completed questionnaire, 20 families participated in interviews, 17 clinicians participated in focus groups.	Parents felt seeking DC at a time point after their child’s stabilising was more appropriate than seeking consent at an earlier, more critical time point, assisted with considering trial information. Practitioners believed such timing assisted informed decision-making.	
Menzies, 2011 [25](Abstract only)	UK PICU	Acceptability of consent processes in emergency research	Qualitative study	Focus groups, 8 adults of 5 children	Parents want to make the decision about their child entering a trial. Deferred consent is only acceptable if there is some form of communication with them at trial entry	Quality not assessed
Rademacher, 2013[39](Abstract only)	USA	Parental attitudes about conducting research of therapies for severe TBI in children using EFIC.	Cross sectional web based survey	1637 parents of children 0–17.	More than a third of parents agree with including children with TBI in research studies when parents are not present for consent, less than half of parents disagree.	Quality not assessed
Scholefield, 2011 [29](Abstract only)	UK	Children’s views on the acceptability of DC	Qualitative study	Interviews, 14 childen (aged 9 to 18)	Trust of the medical profession, and emergency research is safe and therapeutic. Difficulty differentiating between research and clinical decision-making	Quality not assessed
Woolfall, 2016 [30](Abstract only)	UK	Children and young persons views on research without prior consent	Qualitative study	Interviews, 14 children and young people (aged 7 to 15 years)	Supported inclusion in research without prior consent if the trial intervention was thought to be safe and of potential benefit to participants and others. CYP felt that they have the right to be informed and have a say about their participation in a trial as soon as they had recovered	Quality not assessed

*PIS* participant information sheet, *USA* United States of America, *UK* United Kingdom, *TBI* traumatic brain injury, *DC* deferred consent, *IC* informed consent, *RCT* randomised controlled trial, *EFIC* exception from informed consent, *PICU* paediatric intensive care unit

**Table 2:**
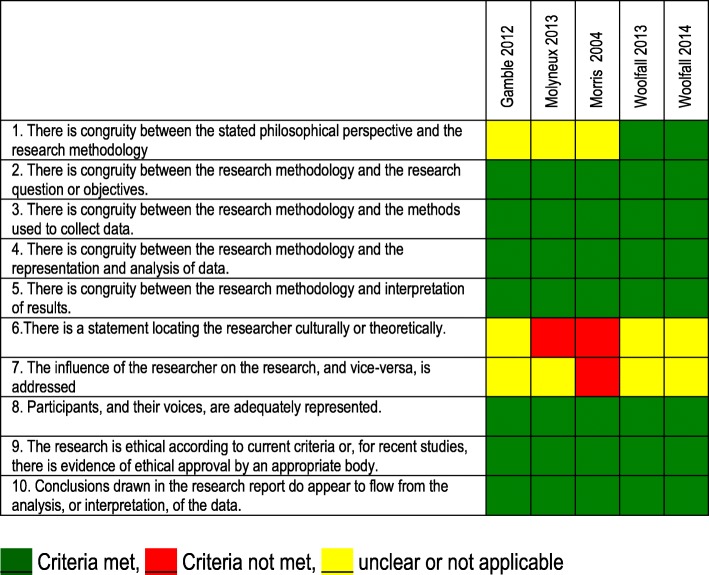
Critical appraisal of qualitative studies

**Table 3:**
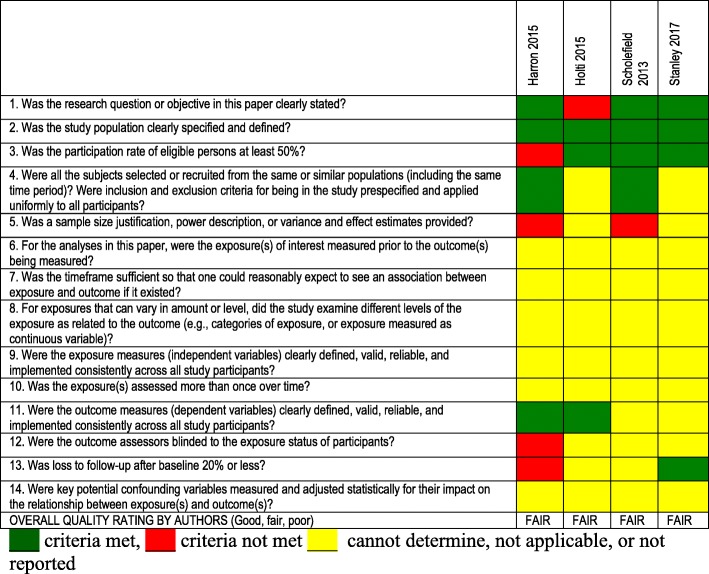
Critical appraisal of quantitative studies

### Capacity of parents or guardians to provide prospective informed consent

The capacity of the consenting individual is a critical requirement to providing valid prospective informed consent. Five quantitative, mixed methods and qualitative studies have provided data on capacity of parents to provide informed consent in the context of emergency and critical care research [19–23].

Practitioners’ perspectives on parental capacity to provide prospective informed consent for a child in the context of emergency and critical care research indicated a divergence of views, largely regarding the ability of laypeople to process and comprehend information at a highly stressful time such as an emergency event [19, 21–23]. Practitioners across the included literature generally reported that parents had a diminished ability to process information and comprehend trial information, especially in the acute stages of a child’s presentation [19, 21–23], and that meaningful consent in these circumstances was not possible [19]. Harron et al., found that some participants were not approached for deferred consent after randomization as research staff were concerned that they were *“not in the right state of mind”* [20]. However, this view was not universal, as in a study by Woolfall et al. 26/45 practitioners believed parents understood trial information provided in an emergency situation *“well”* or *“very well”,* with about one third of those surveyed remaining undecided [22].

Parental perspectives on capacity to provide informed consent were similar to those of practitioners, in terms of diminished ability to process and comprehend information in the face of high stress during the acute care stages of presentation [19, 21, 22]. Supporting this, in a study where deferred or retrospective consent was obtained, parents demonstrated relatively poor comprehension of important research elements and almost a quarter described their experience as clinical care [21].

### Feasibility of prospective informed consent

Two studies specifically addressed whether prospective informed consent was feasible [19, 24]. A study conducted by the Pediatric Emergency Care Applied Research Network (PECARN), exploring the feasibility of various aspects of a study of moderate to severe traumatic brain injury, found that parents and guardians are often not available within the narrow therapeutic window of investigational therapies [24]. While children often arrived within an hour or two of injury, most parents and guardians did not arrive until 2 to 3 h or later. This was more apparent for children transferred from another site and more severely injured children. The authors concluded that an exception of informed consent would be necessary for timely enrolment of children into such a trial [24]. A qualitative study using focus groups of parents and staff of a paediatric intensive care setting to discuss a cardiac arrest research scenario, concluded that meaningful prospective informed consent was not feasible, and endorsed exception of informed consent, with the proviso that parents were offered an opportunity to decline participation prior to enrolment [19].

### Support for alternatives to prospective informed consent

Estimates of support for research with alternatives to informed consent are broad and generally influenced by a number of factors. Five included studies were performed as part of a community consultation process, which is a federal requirement in the USA for research performed under a waiver of informed consent [12], and used in other settings as well [21, 25]. These studies have quantified the level of support; however combining these estimates is inappropriate because of the heterogeneity of methods used and the specific contexts of the individual studies. Community consultation has included perspectives of both the parents of prospective participants, as well as health professionals.

A random dialling phone survey of over 2000 participants, for an out of hospital resuscitation study conducted in 5 states in the USA explored support for the exception to written consent in both adult participants and the 15–17 year old subgroup of the trial [26]. The study found 42.7–71.0% supported the exception to written consent being justified for 15–17 year olds, and in the best interests of the patients and the community which was only slightly lower than support for adults in the same trial [26]. Similar support has been reported in a UK study of parents of children who had suffered bacterial meningitis or meningococcal septicaemia, including bereaved parents [27]. In a postal survey 45/66 (68%) indicated they would be willing for their child to be included in a trial without the trial being explained beforehand [27]. In a study of inpatient resuscitation research, more than 60% of parents were supportive of the study procedures including the exception to prospective informed consent [19].

In qualitative studies parents were generally supportive of research without prior consent [28], with reasons including altruism and general trust in the medical profession to make appropriate decisions [23, 27–30]. However, this sentiment was often accompanied by reservations about the level of risk or potential for harm of the intervention, or as dependent on the type of study being performed [23, 28]. A common theme was the importance to prioritise the management of the child prior to detailed explanations or excessive paperwork [23]. Some parents’ support for research without prospective consent was contingent on the child’s outcome [19, 28]. Such reservations led to an emphasis on the importance of appropriate explanations regarding the necessity for a deferred consent process in these research settings [28].

While the majority of studies have demonstrated that most parents understand and support the concept, some individuals hold strongly opposing views about research without prior consent, taking the perspective that a child should not be exposed to research without prior consent, and parents must be consulted before children are enrolled [23, 27]. Common reasons for opposing research without consent include the fear of adverse effects and feelings that the parents should *“not lose the right to consent”* [26].

The health professionals’ perspective varied in terms of support for research without prior consent. USA researchers found only 50% of staff supported a trial with exception to informed consent. However a large proportion were neutral (38%) and only 12% opposed the planned trial procedures [19]. In the UK, a survey of emergency medicine consultants found that 34/46 (74%) believed deferred consent would be acceptable for a planned trial evaluating therapeutic hypothermia following a paediatric cardiac arrest [31]. Qualitative studies have explored reasoning behind divergent views regarding research without prospective informed consent [19, 22, 23, 28]. Practitioners and researchers enrolling children in studies suggested familiarity with using a deferred consent process influenced acceptability and level of comfort of the procedure. Practitioners and researchers who had previous experience of the deferred consent method generally reported families as being receptive to the method if handled sensitively [22].

Only two identified studies reported the opinions of children on research without prior consent, and both were available in abstract form only [29, 30]. Children in these studies generally regarded the use of exception from informed consent as acceptable [29], especially in life threatening situations [30].

### Community consultation

Two studies explored other issues around community consultation including cost, value and variability in implementation [26, 32]. Requirements of community consultation are at the discretion of local institutional review boards (IRBs) and variability in requirements was evident, particularly when involving multiple centres and different jurisdictions [26, 32]. Methods of community consultation included focus groups, interviews, surveys, town meetings, and public disclosure involving news releases, mailings and public service announcements. Another study with various modalities found focus groups were not well attended, with a quarter having no attendees. Only 5% of research participants had heard about the trial from community consultation and public disclosure activities [32]. The cost of community consultation was reported in two studies. The phone surveys conducted by one large multicentre study averaged US$15,000 per site [26]. Another study utilising various modalities calculated the median cost of activities was about US$7000 [32]. The median additional time of this process was 10 months.

### Process issues

Parents commented on the amount of information provided on consent forms as an issue in decision making [23, 28]. When the child was ill, parents prioritised the treatment of the child over consent procedures, and preferred simple clear information on a single page [23].

A process of pre-consent was considered in two studies where potential participants are given the opportunity to consent or opt-out of participating in a trial, before they meet eligibility criteria, typically in an at risk population [19, 32]. In a study of paediatric status epilepticus, over 4000 patients considered at risk of prolonged seizures received information about the trial, but only 6 out of 208 patients who’s parents were pre-consented were subsequently enrolled in the trial, constituting only 3% of the 310 patients enrolled in the trial [32]. A further 158 parents chose to place their child on the opt out list [32]. In a qualitative study of paediatric cardiac arrest in a paediatric intensive care unit (PICU) setting, pre-consent was perceived as an excessive burden to parents and the validity of consent in this situation was questioned by the authors, as parents may have presumed the study details were not applicable to them at the time of consent, and therefore did not consider the implications adequately [19].

In circumstances where consent is delayed, meaning that the intervention is commenced without consent, but consent sought later to continue with the trial and for the use of data, the timing of approaching parents with trial information is important. Such studies have been variably described as delayed, deferred or retrospective consent. Nine studies specifically used the term “deferred consent” [20–23, 25, 27–29, 31]. Four studies discussed implications concerning the timing of approach for consent when retrospective or deferred consent processes are used [22, 23, 27, 28]. Generally, across parents and practitioners there is agreement that approach for consent in these circumstances should occur once the child’s condition is perceived to be stabilised [22, 23, 27, 28]. Both practitioners and parents expressed views that the timing of the approach, could affect the likelihood of agreeing [22, 23].

### Modified or limited consent process

While acknowledging the difficulties of obtaining prospective informed consent in a number of studies, participants often preferred *“some consent”* rather than enrolment with no information at all [19, 21, 25]. The suggested modified consent usually took the form of brief verbal consent, or “assent” of parents at enrolment [19, 21, 25]. A study of the views parents of children admitted to a PICU about a deferred consent project, found they thought the process was only acceptable if there was some information provided at enrolment [25]. In a study that utilised both full prospective informed consent (when possible) and “assent” in other circumstances, consisting of a single paragraph briefly explaining the trial being read to participants. About half of participants were enrolled with each process overall, however the proportions varied between sites, suggesting physician preference and comfort with procedures, rather than only participant and parent factors influenced the type of consent used [21]. Only 0.4% who assented withdrew consent later. Staff generally supported the process in this setting, however some questioned the validity of assent in these circumstances or thought it too might delay treatment [21].

### Exploring issues of child death during the research

Six studies reported relevant data regarding the situation of child death during research and use of alternatives to prospective informed consent. Issues included whether seeking consent was appropriate, whether consent should be waived in this circumstance and the need to balance the additional burden of disclosure to parents against their right to be informed [27, 28].

Studies of parental opinion regarding the disclosure of participation in research and deferred consent being sought in the case of child death during a trial have found mixed results [27, 28]. Some data suggest the majority of parents favour disclosure, and altruism in that the data could contribute to the greater good, usually stated as a reason [23, 27, 28]. However, contrasting views were also apparent with some parents strongly favouring non-disclosure in this situation [28]. Gamble et al. explored and compared attitudes of bereaved and non-bereaved parents and suggests attitudes were different, with the majority (66%) of bereaved parents favouring disclosure contrasting with 57% of non-bereaved parents expressing a preference for non-disclosure. Preference for non-disclosure was usually to avoid causing additional distress to grieving parents [27].

Two studies reported data from the CATCH trial, where children were enrolled in both emergency and elective settings [20, 23]. Of children enrolled in an emergency setting consent was obtained for only 984/1358 (72%) because of lack of opportunity or because staff decided not to approach parents. Consent was refused for 26 children who died and 151 who survived, but the reasons for refusal differed between groups. The mortality rate of consented children was 9%, compared to 18% for non-consented children, whose data were excluded from analysis [20]. A qualitative evaluation of this trial including bereaved parents, found some were *“shocked”* that their children had been enrolled in research without prior consent [23]. Others described experiences where they thought the manner of approach had been insensitive. Doctors felt that approach after death was far more challenging [23], and clinicians frequently opted to not approach grieving families [20]. A contrasting method was adopted by investigators (and ethics committees) of the FEAST study, who deemed it *“unethical”* to approach parents when a child died, and included data for patients who provided assent and waived the requirement for informed, deferred consent [21]. Opinions varied in relation to the most appropriate time to approach parents for consent in the case of child death during a trial. Mostly, data suggest that approaching bereaved parents for consent should *“not be too soon”* and advocating clinician discretion [27, 28]. Children reportedly understood the potential for bias with refusal of parental consent in a deferred consent study [29].

## Discussion

Our systematic review of stakeholder attitudes to alternatives to prospective informed consent in paediatric emergency medicine found the limited available evidence suggested that children, families and practitioners were aware of the limitations of prospective informed consent for emergency and time critical research, were generally supportive and seemed to acknowledge the requirement for alternative strategies. Identified barriers to informed consent included the capacity of parents, insufficient time (compared to therapeutic windows of interventions), and some process issues like paperwork. Modifications to some processes were proposed.

The diminished capacity of parents to consent under stressful circumstances should not be surprising. Even under ideal circumstances research participants are often demonstrated to have suboptimal understanding [33, 34]. Similarly, in emergency surgery situations the validity of consent for clinical care has been questioned due to poor retention of information [35]. In the research context a concept of the “therapeutic misconception” is a common theme, where it is not clear whether parents can accurately differentiate consent for clinical care and research participation.

The terminology used in studies with research without prospective informed consent differed between studies and international variation was apparent. Some authors have criticised terms such as “deferred”, “delayed” or “retrospective” consent, and contend that consent is not possible after the fact, and contravenes the principle of respect for autonomy [1, 36]. However international guidance documents highlight the requirement for research when consent is not possible, and the importance of discussing the research with the patient or surrogate decision maker as soon as possible in such circumstances [4, 10]. The term deferred consent has been used in the medical literature since the 1990s, and tends to refer to permission to continue in the study, or if the study intervention has ended, permission to use the data [1]. Legislation was specifically introduced in Europe and the UK to allow much needed research to occur in situations where obtaining prior informed consent was not possible, which was identified as a problem under the previous legislative arrangements. The USA has similar legislation, where research needs to meet requirements for the federal “exception from informed consent” [12]. In our review, nine of the included studies specifically addressed, and used the term “deferred consent”, meaning it was the most commonly evaluated strategy when prospective informed consent was not possible [20–23, 25, 27–29, 31]. In the Australian context, while the NHMRC National Statement does not specifically use the term deferred consent, section 4.4.14 reinforces the process of informing participants, with the statement *“As soon as reasonably possible, the participant and/or the participant’s relatives and authorised representatives should be informed of the participants inclusion in the research and the option to withdraw from it without any reduction in quality of care”* [4]. This seems to refer to and seek to achieve similar objectives as a deferred consent process.

While research evaluating alternatives to prospective informed consent has been performed in adults, there is relatively few studies in the paediatric setting. We hypothesized that parents and the general community may be less inclined to support research of this type in children, however the majority of people recognised the need for this research to occur, and supported the requirement for research without prospective informed consent, which was similar to previous adult studies [5]. A major limiting factor was the “situational incapacity” of parents precluding valid consent even if immediately available, and limited time for valid prospective informed consent in many situations.

Alternative strategies were proposed that included the opportunity to consent prior to meeting inclusion criteria, the option to “opt out” at the time of enrolment and versions of a modified consent process [19, 21, 25]. Prior consent is seldom a viable option for emergency research, as prior identification of potentially eligible patients is often not feasible, and efforts for prior consent are usually prohibitively inefficient, and may result in selection bias. In emergency trials, particularly in paediatrics the target population is not easily identified in advance. Community consultation efforts in the USA have often included an “opt out” option for clinical trials conducted under the exception to informed consent legislation, but again the process is inefficient, and difficult to implement, with few patients excluded on this basis [26, 32]. An alternative that may not be applicable in all circumstances is the middle ground, of including a brief verbal consent or “assent” process, prior to enrolment in a trial [21]. In extremely time critical interventions, such as cardiac arrest, delays of just minutes may cause harm, therefore this approach would not be useful, but in other circumstances it may be a viable option and fulfil the parents desire to be involved in decision making, reduce some processes of informed consent like paperwork, focus more on managing the child and importantly given the opportunity to decline participation prior to enrolment.

### Limitations

Our review had a number of limitations. Firstly there is no consensus on how to assess quality in qualitative research, or the utility of such an assessment [37]. Over 100-quality assessment tools have been proposed and used for the purposes of critical appraisal of qualitative studies and several are in relatively common use [38]. We used the Qualitative Assessment and Review Instrument (QARI) from the Joanna Briggs Institute [18], which has been widely used for this purpose, and no studies were excluded on the basis of quality assessment, and no studies were deemed to be of low quality. Abstracts were included in the review, which did not contain sufficient information to allow formal quality assessment. It should be recognised that this review identified only 13 studies, which limits the conclusions that can be made. In particular, data on the perspectives of children were lacking. Implications and conclusions for our setting are also hampered by the absence of any Australian studies. Most included studies were from the USA or UK, which may be somewhat applicable in the Australian context due to a degree of similarity with health systems, societal norms and shared values.

## Conclusion

In conclusion, our systematic review of attitudes of stakeholders on alternatives to prospective informed consent in paediatric emergency research demonstrated that children, their families, health care staff, institutions, and the community seem to recognise the requirement for research performed without prior consent, and are generally supportive of enrolling children in such research with the provisions of limiting the degree of risk, and informing parents and/or children as soon as possible. There is a noted lack of Australian data as well as an insufficient understanding of the perspectives of children; both areas represent important knowledge gaps that need to be addressed through high quality research. Giving patients and their families a voice in discussions of alternatives to informed consent in emergency and critical care research in children, and greater engagement in the design of studies is necessary to maintain the trust of the community, and allow vital research to continue.

## Additional file

Additional file 1: Medline (Ovid) search. (DOCX 109 kb)

## Abbreviations

ED: Emergency departments
IRB: Institutional review boards
NHMRC: National Health and Medical Research Council
PECARN: Pediatric Emergency Care Applied Research Network
PICU: Paediatric intensive care unit
UK: United Kingdom
USA: United States of America
