# Supplementary material for: A comprehensive systematic review of stakeholder attitudes to alternatives to prospective informed consent in paediatric acute care research
Source: BMC Med Ethics. 2018 Nov 20;19:89. doi: 10.1186/s12910-018-0327-9 (PMC6245534; doi:10.1186/s12910-018-0327-9)
Supplement: Supplementary file 1 — Medline (Ovid) search. (DOCX 109 kb) [file 12910_2018_327_MOESM1_ESM.docx]

**Appendices:**

**Medline (Ovid) Search**

1. exp Emergency Medical Services/

2. exp Emergency Medicine/

3. exp Emergency Treatment/

4. ems.mp.

5. emt.mp.

6. emergency services.mp.

7. emergency medical service$.mp. [mp=title, abstract, original title, name of substance word, subject heading word, keyword heading word, protocol supplementary concept word, rare disease supplementary concept word, unique identifier]

8. emergency practitioner$.mp. [mp=title, abstract, original title, name of substance word, subject heading word, keyword heading word,

protocol supplementary concept word, rare disease supplementary concept word, unique identifier]

9. emergency triage.mp. [mp=title, abstract, original title, name of substance word, subject heading word, keyword heading word, protocol

supplementary concept word, rare disease supplementary concept word, unique identifier]

10. emergency care practitioner.mp.

11. exp Physician Assistants/

12. exp Emergencies/

13. emergenc$.mp. [mp=title, abstract, original title, name of substance word, subject heading word, keyword heading word, protocol

supplementary concept word, rare disease supplementary concept word, unique identifier]

14. exp Resuscitation/ or exp Resuscitation Orders/

15. 1 or 2 or 3 or 4 or 5 or 6 or 7 or 8 or 9 or 10 or 11 or 12 or 13 or 14

16. Pediatrics/

17. pediatric*.mp. [mp=title, abstract, original title, name of substance word, subject heading word, keyword heading word, protocol

supplementary concept word, rare disease supplementary concept word, unique identifier]

18. paediatric*.mp. [mp=title, abstract, original title, name of substance word, subject heading word, keyword heading word, protocol

supplementary concept word, rare disease supplementary concept word, unique identifier]

19. peadiatric*.mp. [mp=title, abstract, original title, name of substance word, subject heading word, keyword heading word, protocol

supplementary concept word, rare disease supplementary concept word, unique identifier]

20. exp Minors/

21. minor*.mp. [mp=title, abstract, original title, name of substance word, subject heading word, keyword heading word, protocol supplementary concept word, rare disease supplementary concept word, unique identifier]

22. boy.mp. [mp=title, abstract, original title, name of substance word, subject heading word, keyword heading word, protocol supplementary concept word, rare disease supplementary concept word, unique identifier]

23. boys.mp. [mp=title, abstract, original title, name of substance word, subject heading word, keyword heading word, protocol supplementary concept word, rare disease supplementary concept word, unique identifier]

24. boyfriend.mp. [mp=title, abstract, original title, name of substance word, subject heading word, keyword heading word, protocol

supplementary concept word, rare disease supplementary concept word, unique identifier]

25. boyhood.mp. [mp=title, abstract, original title, name of substance word, subject heading word, keyword heading word, protocol

supplementary concept word, rare disease supplementary concept word, unique identifier]

26. girl*.mp. [mp=title, abstract, original title, name of substance word, subject heading word, keyword heading word, protocol supplementary concept word, rare disease supplementary concept word, unique identifier]

27. kid.mp. [mp=title, abstract, original title, name of substance word, subject heading word, keyword heading word, protocol supplementary concept word, rare disease supplementary concept word, unique identifier]

28. kids.mp. [mp=title, abstract, original title, name of substance word, subject heading word, keyword heading word, protocol supplementary concept word, rare disease supplementary concept word, unique identifier]

29. child.mp. [mp=title, abstract, original title, name of substance word, subject heading word, keyword heading word, protocol supplementary concept word, rare disease supplementary concept word, unique identifier]

30. child*.mp. [mp=title, abstract, original title, name of substance word, subject heading word, keyword heading word, protocol supplementary concept word, rare disease supplementary concept word, unique identifier]

31. children*.mp. [mp=title, abstract, original title, name of substance word, subject heading word, keyword heading word, protocol

supplementary concept word, rare disease supplementary concept word, unique identifier]

32. schoolchild*.mp. [mp=title, abstract, original title, name of substance word, subject heading word, keyword heading word, protocol

supplementary concept word, rare disease supplementary concept word, unique identifier]

33. school child.ab,ti.

34. "school child*".ab,ti.

35. adolescen*.mp. [mp=title, abstract, original title, name of substance word, subject heading word, keyword heading word, protocol

supplementary concept word, rare disease supplementary concept word, unique identifier]

36. juvenil*.mp. [mp=title, abstract, original title, name of substance word, subject heading word, keyword heading word, protocol supplementary concept word, rare disease supplementary concept word, unique identifier]

37. youth*.mp. [mp=title, abstract, original title, name of substance word, subject heading word, keyword heading word, protocol supplementary concept word, rare disease supplementary concept word, unique identifier]

38. teen*.mp. [mp=title, abstract, original title, name of substance word, subject heading word, keyword heading word, protocol supplementary concept word, rare disease supplementary concept word, unique identifier]

39. under*age*.mp. [mp=title, abstract, original title, name of substance word, subject heading word, keyword heading word, protocol

supplementary concept word, rare disease supplementary concept word, unique identifier]

40. pubescen*.mp. [mp=title, abstract, original title, name of substance word, subject heading word, keyword heading word, protocol

supplementary concept word, rare disease supplementary concept word, unique identifier]

41. school.ab,ti.

42. "school*".ab,ti.

43. 16 or 17 or 18 or 19 or 20 or 21 or 22 or 23 or 24 or 25 or 26 or 27 or 28 or 29 or 30 or 31 or 32 or 33 or 34 or 35 or 36 or 37 or 38 or 39 or 40 or 41 or 42

44. exp Informed Consent/

45. deferred.ab,ti.

46. delayed.ab,ti.

47. waiver.ab,ti.

48. exception.ab,ti.

49. retrospective.ab,ti.

50. alternative.ab,ti.

51. 45 or 46 or 47 or 48 or 49 or 50

52. 44 and 51

53. (deferred adj3 consent).mp. [mp=title, abstract, original title, name of substance word, subject heading word, keyword heading word, protocol supplementary concept word, rare disease supplementary concept word, unique identifier]

54. (delayed adj3 consent).mp. [mp=title, abstract, original title, name of substance word, subject heading word, keyword heading word, protocol supplementary concept word, rare disease supplementary concept word, unique identifier]

55. (waiver adj3 consent).mp. [mp=title, abstract, original title, name of substance word, subject heading word, keyword heading word, protocol supplementary concept word, rare disease supplementary concept word, unique identifier]

56. (exception adj3 consent).mp. [mp=title, abstract, original title, name of substance word, subject heading word, keyword heading word,

protocol supplementary concept word, rare disease supplementary concept word, unique identifier]

57. (retrospective adj3 consent).mp. [mp=title, abstract, original title, name of substance word, subject heading word, keyword heading word, protocol supplementary concept word, rare disease supplementary concept word, unique identifier]

58. (alternative adj3 consent).mp. [mp=title, abstract, original title, name of substance word, subject heading word, keyword heading word, protocol supplementary concept word, rare disease supplementary concept word, unique identifier]

59. 53 or 54 or 55 or 56 or 57 or 58

60. 52 or 59

61. 15 and 43 and 60
